# Association Between Periodontitis and Cognitive Impairment in Adults: A Systematic Review

**DOI:** 10.3389/fneur.2019.00323

**Published:** 2019-04-24

**Authors:** Priscila Cunha Nascimento, Micaele Maria Lopes Castro, Marcela Baraúna Magno, Anna Paula Costa Ponte Sousa Carvalho Almeida, Nathália Carolina Fernandes Fagundes, Lucianne Cople Maia, Rafael Rodrigues Lima

**Affiliations:** ^1^Laboratory of Functional and Structural Biology, Institute of Biological Sciences, Federal University of Para, Belém, Brazil; ^2^Department of Pediatric Dentistry and Orthodontics, School of Dentistry, Federal University of Rio de Janeiro, Rio de Janeiro, Brazil; ^3^School of Dentistry, Faculty of Medicine and Dentistry, University of Alberta, Edmonton, AB, Canada

**Keywords:** periodontitis, cognitive impairment, dementia, systematic review, oral health, cognitive dysfunction

## Abstract

Periodontitis is an oral inflammatory disease and may contribute to low-grade systemic inflammation. Based on the contribution of periodontitis to systemic inflammation and the potential role of systemic inflammation in neuroinflammation, many epidemiological studies have investigated a possible association between periodontitis and mild cognitive impairment or dementia. The purpose of this study was to evaluate the clinical/epidemiological evidence regarding the association between periodontitis and cognitive decline in adult patients. A search conducted between September and October 2018 was performed in the electronic databases PubMed, Scopus, Web of Science, The Cochrane Library, LILACS, OpenGrey, and Google Scholar, with no publication date or language restrictions. Analytical observational studies in adults (P—Participants), with (E—Exposure) and without periodontitis (C—Comparison) were included in order to determine the association between periodontitis and cognitive decline (O—Outcome). The search identified 509 references, of which eight observational studies were accorded with the eligibility criteria and evaluated. The results should, however, be interpreted cautiously due to the limited number of studies. This systematic review points to the need for further well-designed studies, such as longitudinal observational studies with control of modifiable variables, as diagnostic criteria and time since diagnosis of periodontitis and cognitive impairment, to confirm the proposed association.

## Introduction

As life expectancy has increased and the elderly population has grown, there has been an increase in the prevalence of age-related diseases, as well as mild cognitive impairment (MCI) (1–5). The MCI is a pathological cognitive state with potential progression to dementia, resulting in social and health sequelae (6). According to the World Health Organization (WHO), the number of people living with dementia is expected to triple, from 50 to 152 million by 2050, which is alarming as it is one of the leading causes of mortality and disability in elderly adults (7, 8).

Given the increasing prevalence of age-related diseases, investigating modifiable risk factors for dementia is an essential objective for developing preventative strategies. The known risk factors for dementia include age, presence of apolipoprotein E allele (9), family history and schooling (10–13). Chronic inflammation has also been identified as a risk factor on this disease (14–16).

Periodontitis is a chronic inflammatory and infectious disease of multifactorial etiology, which affects the protective and support tissue of teeth, the periodontium (17). This is the most prevalent disease in the oral cavity, as well as dental caries (18, 19). Studies have proposed that periodontitis induces chronic systemic inflammation, which stimulates the production of inflammatory cytokines, among them interleukin 1β (IL-1β), interleukin 6 (IL-6) and tumor necrosis factor-alpha (TNF-α), and contributes to an increase in the neuroinflammatory response (20–24).

Periodontitis may be associated with other chronic inflammatory systemic diseases (25), including cardiovascular diseases (26) such as atherosclerosis (27), rheumatoid arthritis (28) and diabetes (29). This evidence suggests that periodontitis is an important source of inflammatory mediators reaching all systems through the blood circulation (24). Based on the contribution of periodontitis to systemic inflammation and the potential role of systemic inflammation in neuroinflammation (24), it is relevant to consider an association between chronic periodontitis and cognitive impairment. The aim of this study was, through a systematic review to analyze the association between periodontitis and cognitive impairment in adults.

## Methods

### Protocol and Registration

This systematic review was recorded in the PROSPERO database (https://www.crd.york.ac.uk/PROSPERO/), under registration number CRD42017056276. The PRISMA guidelines were followed (Supplementary Table S1) (30, 31).

### Study Design and Eligibility Criteria

After the initial search and exclusion of duplicate records, studies were selected for full reading based on the acronyms PECO from the evaluation of the title and abstract. We included observational studies focused in adult humans (P) in which patients with periodontitis (E) and without periodontitis (C) were part of the same investigation, in order to observe an association between periodontitis and cognitive decline (O). This strategy aimed to answer the following question: Is there an association between periodontitis and cognitive impairment in adults?

The selection of articles was also based on the following exclusion criteria: studies that reported intervention, such as periodontal therapy and patients with gingivitis only (periodontal inflammation without bone loss), as well as review articles, case reports, letters, editorials, theses or articles that did not include the results of interest.

### Search Strategy

The literature review was conducted in the following electronic databases: PubMed, Scopus, Web of Science, The Cochrane Library and LILACS. The gray literature was consulted through OpenGrey and Google Scholar. The search was conducted between September and October 2018. Data collection was done independently by two reviewers (PCN, MMCL). Any disagreements between the examiners were resolved by a third reviewer (RRL).

The MeSH terms, keywords and detailed search strategies were appropriately adapted for each database using the Boolean operators (OR, AND) to combine the searches (Supplementary Table S2).

No restrictions were imposed on the date of publication or the language used in primary studies. A manual search was performed on the reference lists of included studies to find additional studies. An alert was created on each database for the new studies included after the running of searches. Articles that were in more than one database were considered only once.

### Data Extraction

After collecting the references in the databases, all articles were stored in a reference manager (EndNote®, version X7, Thomson Reuters). Initially, we exclude duplicates, leaving the articles for analysis by title and abstract following the eligibility criteria. From then on, the articles elected were analyzed and judged in their full-text.

After selection, the following data were extracted from the included articles in the qualitative analysis: year, source of sample; characteristics of the participants (sample size and age); evaluation methods (for periodontitis and cognitive decline); statistical analysis, results and outcomes.

### Data Analysis

#### Risk of Bias

For the evaluation of the methodological quality and the risk of bias, the checklist of Fowkes and Fulton (32) was applied. This checklist has domains covering the study and sample design; characteristics of the control group; quality of measures and results; and integrity and distorting influences.

After evaluating each criterion, different signals were attributed in cases of major problems (++) in the study or in minor problems (+), in order to evaluate if the methods are adequate to produce consistent and valid information, as well as if the results expected effects that could draw conclusions. In the items where the question was not applicable to the type of study, the acronym NA (not applicable) was assigned. No problem was assigned using another signal (0). The evaluation for each domain was standardized by an adapted version to the criteria previously described (Supplementary Table S3) (27).

After a detailed evaluation of the methods and results, the studies were analyzed to verify the possibility of “biased results,” “confusions” and “chance occurrence.” In order to determine the value of the study, three summary questions were answered: “Are the results erroneously biased in certain direction?”; “Are there any serious confusing or other distorting influences?” and “Is it likely that the results occurred by chance?” “YES” and “NO” answers were assigned. If the answer is NO in the three questions, the article is considered reliable, with low risk of bias.

## Results

### Study Selection

A total of 509 relevant studies were identified in the electronic databases. Among these, 108 were duplicates and were excluded. Of the remainers, 401 titles and abstracts were analyzed and 390 were eliminated as they did not satisfy the inclusion criteria. The full text evaluation results in 11 articles (33–43).

**Figure 1:**
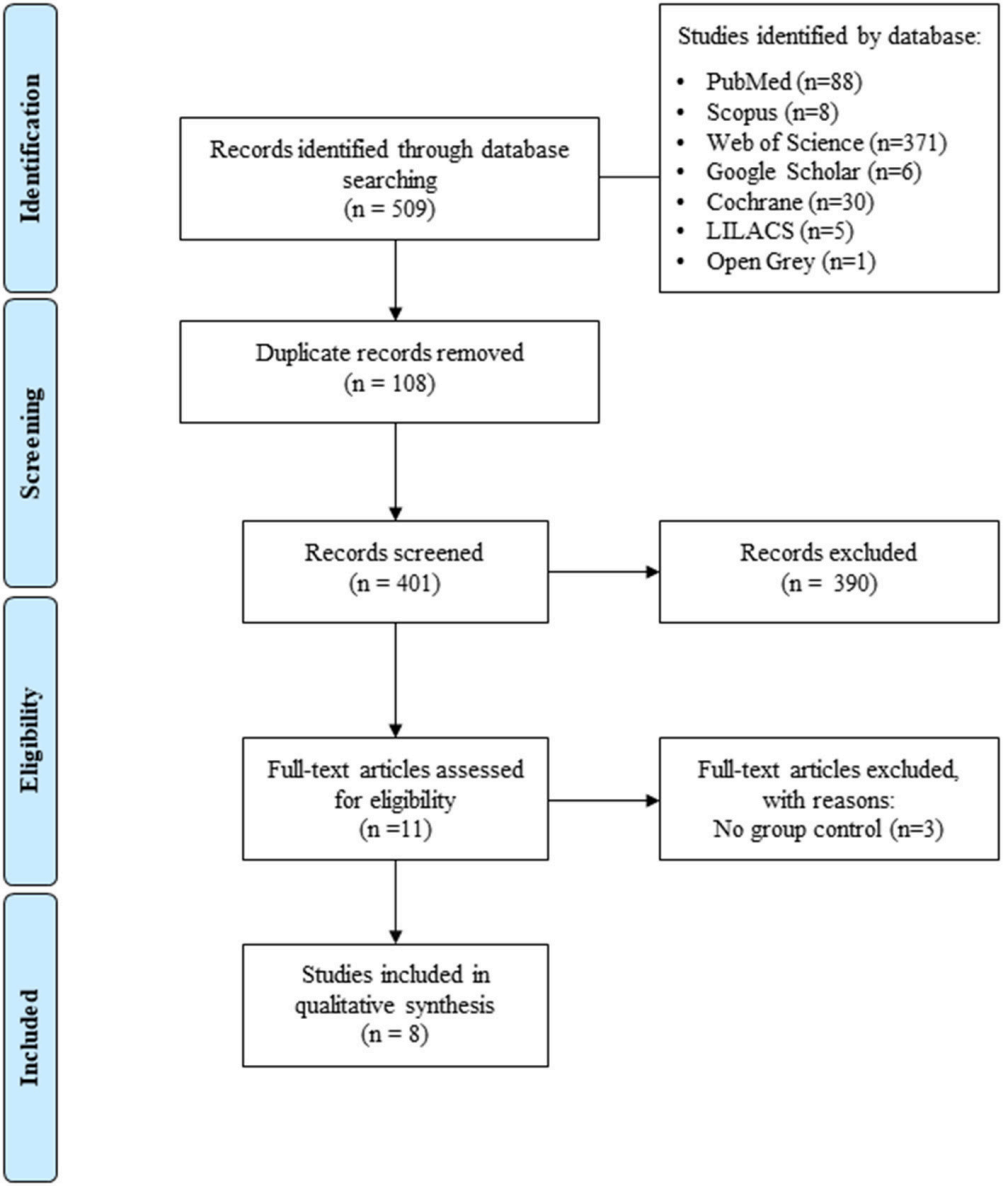
Flow diagram of databases was searching accordingly with PRISMA guidelines (30).

Three articles (36, 37, 39) were subsequently excluded due to the lack of a control group (adult patients without periodontitis). Thus, eight studies were included in the qualitative synthesis (33–35, 38, 40–43). All steps were performed by two reviewers (PCN and MMLC) and checked by a third reviewer in the case of disagreement (RRL). Each step is described in Figure 1 according to the PRISMA flowchart (30).

### Characteristics of the Studies

In the eight articles included, the samples were from communities, hospitals, and national epidemiological surveys. One of them (43) was classified as a prospective cohort, four studies (35, 40–42) were retrospective cohorts and three were transverse studies (33, 34, 38). A summary and the characteristics of each study are presented in Table 1.

**Table 1:** Summary of characteristics and results of the included studies.

**Author (year)**	**Study design**	**Participants**		**Diagnostic method**		**Statistical analysis**	**Results**	**Outcome**
		**Source**	**Sample size (*n*) Age**	**Periodontitis**	**Cognitive impairment**			
Chen et al. (42)	Retrospective cohort	National Health Insurance Research Database (NHIRD), Taiwan, 1997–2004	With periodontitis: 9.291 without periodontitis: 18.672 ≥ 50 years	Clinical: ICD-9-CM diagnostic criteria. Code 523.4 (chronic periodontitis)	Clinical: ICD-9-CM diagnostic criteria. Code 331.0 (Alzheimer's Disease)	Cox regression: Analysis—Hazard Ratios (HRs) and 95% confidence intervals (CI)	Adjusted HR: without periodontitis: reference; with periodontitis: 1.707 (CI: 1.152–2.528); *p* = 0.0077	10-year chronic periodontitis exposure was associated with a 1.707-fold increase in the risk of developing AD
Iwasaki et al. (38)	Cross-sectional study	Tosa, Kochi, Japan	183 ≥ 75 years	Clinical: attachment loss (AL): interproximal AL ≥ 5mm in ≥50% of teeth	Clinical: Mini-Mental State Examination (MMSE), low scorebaixo = 23/24; Hasegawa Dementia Scale-Revised, low scorebaixo = 20/21 (HDS-R)	Logistic regression analyses—odds ratios (OR) and 95% confidence intervals (CI)	The multivariable-adjusted OR (CI) for low MMSE score associated with periodontal disease was 2.21 (1.01–4.84) and for low HDS-R the score associated with periodontal disease was 4.85 (1.29–18.15)	Poor oral health status was significantly associated with cognitive impairment among community-dwelling older Japanese
Iwasaki et al. (40)	Retrospective cohort	Tosa, Kochi, Japan	With periodontitis: 21 without periodontitis: 64 > 75 years	Clinical: ≥2 interproximal sites with clinical attachment loss Oz ≥ 6 mm (not on the same tooth) and ≥1 interproximal site with probing depth ≥ 5 mm	Clinical: Mini-Mental State Examination (MMSE)	1. Multivariable Poisson regression analyses—relative risk (RR)—and 2. multivariable linear regression (coefficient), both whit 95% confidence intervals (CI)	1. Periodontitis was significantly associated with an increased risk of cognitive decline [adjusted RR = 2.2 (CI: 1.1 −4.5)]. 2. Participants with severe periodontitis had a 1.8-point greater decrease (CI: 3.3–0.2) in MMSE score than those without severe periodontitis	Periodontitis was significantly associated with a future decline in cognitive function among community-dwelling older Japanese
Kamer et al. (35)	Retrospective cohort	Copenhagen County Hospital and the County Mental Hospital in Glostrup, Copenhagen, Denmark	152 ≥ 70 years	Clinical: inspection and probing using the Modified Community Periodontal Index (MCPI) (44) for the classification of periodontitis	Clinical: Wechsler Adult Intelligence Scale (WAIS), which measures several domains of cognitive function: digit symbol (DST [score 0–90]), picture completion, and block design (BDT [score 0–48])	Logistic regression analyses—odds ratios (OR) and 95% confidence intervals (CI)	Presence of periodontal infection (PI) was strongly associated with having a low DST score (adjusted OR: 7.00; CI: 1.74–28.16)	Subjects with PI obtained lower mean DST scores compared to subjects without PI
Lee et al. (43)	Prospective cohort	National Health Insurance Research Database (NHIRD), Taiwan	With periodontitis: 3,028 without periodontitis: 3,028 ≥ 65 years	Clinical: ICD-9-CM diagnostic criteria. Code 523.3–5 (periodontitis)	Clinical: ICD-9-CM diagnostic criteria. Codes 290.0–290.4, 294.1, 331.0–331.2. (dementias)	Cox proportional hazards regression model—hazard ratios (HR) and 95% confidence intervals (CI)	Participants with periodontitis had a significantly higher risk of developing dementia than controls (adjusted HR 1.16, CI: 1.01–1.32)	Periodontitis was associated with greater risk of developing dementia
Noble et al. (34)	Cross-sectional study	Third National Health and Nutrition Examination Survey (NHANES-III), EUA, 1991–1994	2,355 ≥ 60 years	Laboratory samples (reported in ELISA units of IgG [EU]): Mean *P. gingivalis* IgG for healthy individuals ≤ 57 EU, mild periodontitis 58–65 EU, moderate periodontitis 66–119 EU and severe periodontitis > 119 EU.	Clinical: 1) an immediate and delayed logical verbal memory test from the East Boston Memory Test; 2) a three-word registration/memory task (“apple,” “table” and “penny”); and 3) five serial subtractions by intervals of three.	Logistic regression analyses—odds ratios (OR) and 95% confidence intervals (CI)	Individuals with higher serum levels of IgG for *P. gingivalis* (>119) were more likely to present a delayed verbal recovery (OR 2.89, CI: 1.14–7.29) and impaired (OR 1.95, CI 1.22 to 3.11) then those with lower levels (≤57)	The serological marker of periodontitis is associated with impaired delayed memory and calculation
Tzeng et al. (41)	Retrospective cohort	National Health Insurance Research Database (NHIRD), Taiwan, 2000–2010	With periodontitis: 2,207 without periodontitis: 6,621 ≥ 20 years	Clinical: ICD-9-CM diagnostic criteria. Code: 523.1 (chronic gingivitis) and 523.4 (chronic periodontitis)	Clinical: ICD-9-CM diagnostic criteria. Code: 290.0, 290.10–290.13, 290.20–290.21, 290.3, 290.41–290.43, 290.8–290.9, and 331.0. (dementias)	Cox proportional hazards regression model—hazard ratios (HR) and 95% confidence intervals (CI)	Study subjects were more likely to develop dementia (adjusted HR 2.54, CI: 1.297–3.352)	Patients with chronic periodontitis and gingivitis have a higher risk of developing dementia
Yu et al. (33)	Cross-sectional study	Third National Health and Nutrition Examination Survey (NHANES-III), EUA, 2001–2002	803 ≥ 60 years	Clinical: probing depths in millimeters and clinical attachment level (CAL).	Clinical: 2-minute Digit Symbol Substitution Test (DSST), score 1-133	Logistic regression analyses—odds ratios (OR) and 95% confidence intervals (CI)	Higher cognitive function (higher scores in DSST) was associated with lower odds of periodontal disease (adjusted OR 0.69, CI: 0.51–0.94)	Higher cognitive function was associated with lower odds of periodontal disease in non-institutionalized older adults

*ICD-9-CM: International Classification of Diseases, Ninth Revision, Clinical Modification; P. gingivalis: Poryphyromonas gingivalis*.

Most of the studies (33–35, 38, 40, 42, 43) included adults over 50 years old; only one study (41) included participants of 20 years and older.

For the periodontitis evaluation, most studies considered the following clinical parameters: two articles (33, 40) measured probing depth (mm) and clinical attachment loss; two articles (35, 38) evaluated only probing depth; three articles (41–43) included participants with periodontitis diagnosed according to the International Classification of Diseases, Ninth Revision, Clinical Modification (ICD-9-CM); and one study (34) used laboratory parameters, such as serum levels of immunoglobulins G (IgG) against *Porphyromonas gingivalis (P. gingivalis)*.

The diagnostic tools used to assess cognitive impairment varied among the studies. Three studies (41–43) used diagnostics established by means of ICD-9-CM coding; one study used the Mini-mental State Examination (MMSE) (40), one article (38) used the MMSE with the Hasegawa Dementia Scale-Revised (HDS-R); one study (35) used two Wechsler Adult Intelligence Scale (WAIS) subtests i.e., the Digit Symbol Test (DST) and the Block Design Test (BDT); one study (33) applied the 2 min Digit Symbol Substitution Test (DSST); and one article (34) used three tests: delayed logical verbal memory test from the East Boston Memory Test, three word registration/memory tasks and five serial subtractions by intervals of three.

All articles (33–35, 38, 40–43) highlighted an association between periodontitis and cognitive impairment. Individuals with periodontitis reported a higher probability of developing cognitive decline or an associative relationship between both conditions.

### Risk of Bias

The risk of bias evaluation was performed using three questions associated with the risk of bias, confounding factors and chance occurrence, which summarized the quality assessment of the included studies. Although some studies presented minor problems, they did not influence the final risk of bias, and all were considered as a low risk, with consistent information, as shown in Table 2.

**Table 2:** Quality assessment of studies included, according to Fowkes and Fulton (32).

**Diretriz**	**Lista de verificação**	**Chen et al. (42)**	**Iwasaki et al. (38)**	**Iwasaki et al. (40)**	**Kamer et al. (35)**	**Lee et al. (43)**	**Noble et al. (34)**	**Tzeng et al. (41)**	**Yu et al. (33)**
Study sample representative?	Source of sample	0	0	0	0	0	0	0	0
	Sampling method	0	0	0	0	0	0	0	0
	Sample size	+	+	+	+	+	+	+	+
	Entry criteria/exclusion	0	0	0	0	0	0	0	0
	Non-respondents	0	0	0	0	0	0	0	0
Control group acceptable?	Definition of controls	0	0	0	0	0	0	0	0
	Source of controls	0	0	0	0	0	0	0	0
	Matching/randomization	0	+	+	+	0	0	0	0
	Comparable characteristics	0	0	0	0	0	0	0	0
Quality of measurements and outcomes?	Validity	+	0	0	0	+	+	+	0
	Reproducibility	0	0	0	0	0	0	0	0
	Blindness	0	++	++	++	0	++	0	++
	Quality control	0	0	0	0	0	0	0	0
Completeness	Compliance	0	0	0	0	0	0	0	0
	Drop outs	0	NA	0	0	0	NA	0	NA
	Deaths	0	NA	0	0	0	NA	0	NA
	Missing data	0	NA	0	0	0	NA	0	NA
Distorting influences?	Extraneous treatments	0	0	0	0	0	0	0	0
	Contamination	NA	NA	NA	NA	NA	NA	NA	NA
	Changes over time	0	0	0	0	0	0	0	0
	Confounding factors	0	0	0	0	0	0	0	0
	Distortion reduced by analysis	0	0	0	0	0	0	0	0
Summary questions	Bias:								
	Are the results erroneously biased in certain direction?	NO	NO	NO	NO	NO	NO	NO	NO
	Confounding:								
	Are there any serious confusing or other distorting influences?	NO	NO	NO	NO	NO	NO	NO	NO
	Chances								
	Is it likely that the results occurred by chance?	NO	NO	NO	NO	NO	NO	NO	NO

*0: No problem; +: Minor problem; ++: Major problem; NA: Not Applicable*.

All articles presented a minor problem (+) regarding the item “Sample size” i.e., they did not report a sample size calculation. Likewise, Iwasaki et al. (38), Iwasaki et al. (40) and Kamer et al. (35) did not describe randomization, but reported the matching of groups.

The studies performed by Chen et al. (42), Lee et al. (43) and Tzeng et al. (41) were considered as having a minor problem in the item “Validity,” C as they were based on a diagnosis established by ICD-9-CM coding. This approach presents low specificity due to the absence of clinical parameters or complementary exams in the diagnosis of periodontitis. The study by Noble et al. (34) was also scored as presenting a minor problem (+) in the item “Validity” due to the use of laboratory parameters as the only method to diagnose periodontitis.

Finally, the articles by Iwasaki et al. (38), Iwasaki et al. (40), Noble et al. (34) and Yu and Kuo (33) did not emphasize the blindness between evaluators and the subjects of the research.

## Discussion

This systematic review presents evidence that periodontitis patients may have a higher probability of developing cognitive impairment than those without periodontitis, or suggesting a positive association between the pathologies. This observation is based on eight studies (33–35, 38, 40–43).

Systematic reviews involve the application of methodological strategies that limit the risk of bias, evaluate and summarize crucial scientific evidence. Such systematic analyses can help professionals leaving them up to date for clinical decision-making (45). The quality assessment tools vary according to the type of study (46).

In this systematic review, the included studies were observational analytical studies: one prospective cohort (43); four retrospective cohort (35, 40–42) and three transversal studies (33, 34, 38) which make Fowkes and Fulton (32) checklist valid for the evaluation of methodological quality and risk of bias.

Regarding the outcome, the relationship between periodontitis and cognitive impairment demonstrated by all articles (33–35, 38, 40–43) can be attributed to three possible mechanisms: (1) a direct process through the bloodstream; (2) an indirect process through inflammatory mediators; and (3) induction of expression of platelet aggregation proteins (24, 47).

Periodontitis is an inflammatory disease that affects the periodontium, causing the progressive loss of dental support tissues, with the bacterial plaque having been identified as its primary cause (18, 48). The first possible mechanism linking periodontitis and cognitive impairment suggests that bacteria or viruses present in dental plaque enter into the bloodstream, resulting in a systemic infection and causing direct deleterious effects. From the bloodstream, the pathogens' products pass through the blood-brain barrier and reach the brain, which can directly contribute to cognitive decline, resulting in dementia and subsequently neurodegenerative diseases, such as Alzheimer's Disease (AD) (47, 49).

The second mechanism proposes an association through an indirect process, based on an inflammatory response against periodontitis. The presence of periodontal pathogens initiates an increase in the serum levels of inflammatory mediators such as cytokines (interleukin 6 [IL-6] and tumor necrosis factor alpha [TNF-α]), proteins (C-reactive protein [CRP]) and cell adhesion molecules (Intercellular adhesion molecule [ICAM-1] and vascular cell adhesion molecule [VCAM-1]) (47, 50, 51).

The third mechanism is based on studies that have investigated the expression of platelet aggregation proteins resulting from the presence of periodontal bacteria, including *Streptococcus sanguis* and *P. gingivalis* (47, 52). As a result, periodontitis may play a role in the formation of atheroma and thrombi, contributing to atherosclerotic disease and increasing the risk of cognitive decline due to vascular dementia (53).

Based on the proposed models, this systematic review searched for studies that investigated the association between periodontitis and cognitive impairment. To prove this association, methods of appropriate sensitivity and good specificity should be used. Most of the studies (33, 35, 38, 40) included in this systematic review used evaluation methods validated by the scientific literature, such as probing depth (PD) and clinical attachment loss (CAL). In contrast, three studies (41–43) have used only ICD-9-CM as a parameter to identify periodontitis, without reporting periodontal clinical parameters in this evaluation, which may lead to a questionable clinical validity (54). Noble et al. (34), only used laboratory analysis of a serological marker for *P. gingivalis*. This methodological failure to use the ideal instrument for the diagnosis of periodontitis was what characterized the studies by Chen et al. (42), Lee et al. (43), Tzeng et al. (41) and Noble et al. (34) as having a lower risk (+) in the quality assessment.

Besides using an appropriate diagnostic method, the study sample should be representative to ensure an inferential value. None of the studies included in this systematic review presented a sample size calculation, which can be viewed as a minor problem (+). However, five studies (33, 34, 41–43) obtained their sample from a National Survey, and such surveys are usually based on a prior sample size calculation.

Although the included studies suggests a positive association between periodontitis and cognitive impairment, some limitations were identified in this review. One of these problems was the use of different methods to evaluate cognitive impairment, as the inadequate description of the source of bias, including the size sample calculation, and the lack of reproducibility in the of periodontitis and cognitive impairment diagnosis when using the ICD-9-CM coding parameters. Another limitation was the impossibility to summarize the data in a quantitative analysis. Although the systematic review included 8 studies according to our eligibility criteria, the quantitative synthesis would be inconsistent not only for the diversity among the periodontal clinical parameters and diagnosis of cognitive impairment but also for the presence of a few studies to a group which have these specific parameters.

Based on the results presented by included studies, we cannot rule out the hypothesis that periodontitis and dementia, as common diseases to older adults and/or presenting common risk factors (55), have an eventuality connection. One of the possible reasons for the highlighted association between periodontitis and cognitive impairment is the reverse causality of the two conditions. Individuals with cognitive decline may present deficits in self-care and oral hygiene, which can lead to a higher incidence of periodontitis in these patients (56). The included studies may not answer this association as a result of a pathophysiological process due to a possible insufficient duration of the studies. In addition, the exclusion criteria used by some studies (41–43) may not have been sufficient to ensure that the development of periodontitis preceded the onset of dementia.

## Conclusion

In view of the scientific evidence, an association between periodontitis and cognitive decline is suggested. The diagnosis and treatment of chronic periodontitis is essential for reducing the bacterial source capable of inducing an increase in the levels of proinflammatory cytokines, and, consequently, promoting a better quality of life, especially for elderly adults. There is a need for more observational studies on this topic with control of modifiable variables (diagnostic criteria, time of diagnosis and follow-up between periodontitis and cognitive decline, level of education, etc.) to investigate the cause-effect relationship between the pathologies.

## Author Contributions

PN, AA, NF, LM, and RL: study concept and design. PN, MC, NF and MM: analysis and interpretation of data. PN, MC, and AA: preparation of manuscript. LM and RL: critical revision of manuscript.

### Conflict of Interest Statement

The authors declare that the research was conducted in the absence of any commercial or financial relationships that could be construed as a potential conflict of interest.
