# Supplementary material for: Association Between Periodontitis and Cognitive Impairment in Adults: A Systematic Review
Source: Front Neurol. 2019 Apr 24;10:323. doi: 10.3389/fneur.2019.00323 (PMC6492457; doi:10.3389/fneur.2019.00323)
Supplement: Supplementary file 1 [file Data_Sheet_1.docx]

**Table Supplementary S1.** PRISMA Checklist (Moher et al., 2009).

| **Section/topic** | **#** | **Checklist item** | **Reported on page #** |
| --- | --- | --- | --- |
| **TITLE** | | |  |
| Title | 1 | Identify the report as a systematic review, meta-analysis, or both. | 1 |
| **ABSTRACT** | | |  |
| Structured summary | 2 | Provide a structured summary including, as applicable: background; objectives; data sources; study eligibility criteria, participants, and interventions; study appraisal and synthesis methods; results; limitations; conclusions and implications of key findings; systematic review registration number. | 2 |
| **INTRODUCTION** | | |  |
| Rationale | 3 | Describe the rationale for the review in the context of what is already known. | 3 |
| Objectives | 4 | Provide an explicit statement of questions being addressed with reference to participants, interventions, comparisons, outcomes, and study design (PICOS). | 3 |
| **METHODS** | | |  |
| Protocol and registration | 5 | Indicate if a review protocol exists, if and where it can be accessed (e.g., Web address), and, if available, provide registration information including registration number. | 4 |
| Eligibility criteria | 6 | Specify study characteristics (e.g., PICOS, length of follow-up) and report characteristics (e.g., years considered, language, publication status) used as criteria for eligibility, giving rationale. | 4 |
| Information sources | 7 | Describe all information sources (e.g., databases with dates of coverage, contact with study authors to identify additional studies) in the search and date last searched. | 4 |
| Search | 8 | Present full electronic search strategy for at least one database, including any limits used, such that it could be repeated. | 4 (Supplementary Table S2) |
| Study selection | 9 | State the process for selecting studies (i.e., screening, eligibility, included in systematic review, and, if applicable, included in the meta-analysis). | 4 |
| Data collection process | 10 | Describe method of data extraction from reports (e.g., piloted forms, independently, in duplicate) and any processes for obtaining and confirming data from investigators. | 5 |
| Data items | 11 | List and define all variables for which data were sought (e.g., PICOS, funding sources) and any assumptions and simplifications made. | 4 |
| Risk of bias in individual studies | 12 | Describe methods used for assessing risk of bias of individual studies (including specification of whether this was done at the study or outcome level), and how this information is to be used in any data synthesis. | 5 |
| Summary measures | 13 | State the principal summary measures (e.g., risk ratio, difference in means). | 6 |
| Synthesis of results | 14 | Describe the methods of handling data and combining results of studies, if done, including measures of consistency (e.g., I^2^) for each meta-analysis. | NA |

| **Section/topic** | **#** | **Checklist item** | **Reported on page #** |
| --- | --- | --- | --- |
| Risk of bias across studies | 15 | Specify any assessment of risk of bias that may affect the cumulative evidence (e.g., publication bias, selective reporting within studies). | 5 |
| Additional analyses | 16 | Describe methods of additional analyses (e.g., sensitivity or subgroup analyses, meta-regression), if done, indicating which were pre-specified. | NA |
| **RESULTS** | | |  |
| Study selection | 17 | Give numbers of studies screened, assessed for eligibility, and included in the review, with reasons for exclusions at each stage, ideally with a flow diagram. | 6-7 |
| Study characteristics | 18 | For each study, present characteristics for which data were extracted (e.g., study size, PICOS, follow-up period) and provide the citations. | 8 |
| Risk of bias within studies | 19 | Present data on risk of bias of each study and, if available, any outcome level assessment (see item 12). | 7-8 |
| Results of individual studies | 20 | For all outcomes considered (benefits or harms), present, for each study: (a) simple summary data for each intervention group (b) effect estimates and confidence intervals, ideally with a forest plot. | 7-8 |
| Synthesis of results | 21 | Present results of each meta-analysis done, including confidence intervals and measures of consistency. | 8 |
| Risk of bias across studies | 22 | Present results of any assessment of risk of bias across studies (see Item 15). | 8-9 |
| Additional analysis | 23 | Give results of additional analyses, if done (e.g., sensitivity or subgroup analyses, meta-regression [see Item 16]). | NA |
| **DISCUSSION** | | |  |
| Summary of evidence | 24 | Summarize the main findings including the strength of evidence for each main outcome; consider their relevance to key groups (e.g., healthcare providers, users, and policy makers). | 10 |
| Limitations | 25 | Discuss limitations at study and outcome level (e.g., risk of bias), and at review-level (e.g., incomplete retrieval of identified research, reporting bias). | 12 |
| Conclusions | 26 | Provide a general interpretation of the results in the context of other evidence, and implications for future research. | 12 |
| **FUNDING** | | |  |
| Funding | 27 | Describe sources of funding for the systematic review and other support (e.g., supply of data); role of funders for the systematic review. | 10,12 |

NA: Not Applicable.

**Table Supplementary S2.** Terms used on database search

| Database | Search format |
| --- | --- |
| PUBMED | #1 AND #2 |
|  | #1 ((((((((((((((((((Humans[MeSH Terms]) OR Humans[Title/Abstract]) OR “Man, Modern”[Title/Abstract]) OR “Modern Man”[Title/Abstract]) OR “Man (Taxonomy)”[Title/Abstract]) OR “Homo sapiens”[Title/Abstract]) OR Human[Title/Abstract]) AND Adult[MeSH Terms]) OR Adult[Title/Abstract]) OR Adults[Title/Abstract]) AND Cognition[MeSH Terms]) OR Cognition[Title/Abstract]) OR Cognitions[Title/Abstract]) OR “Cognitive Function”[Title/Abstract]) OR “Cognitive Functions”[Title/Abstract]) OR “Function, Cognitive”[Title/Abstract]) OR “Functions, Cognitive”[Title/Abstract]) AND Memory[MeSH Terms]) OR Memory[Title/Abstract] |
|  | #2 (((((((((((((((((((((((((((((((((((((((((((((((((Periodontitis[MeSH Terms]) OR Periodontitis[Title/Abstract]) OR “Periodontal Diseases”[MeSH Terms]) OR “Periodontal Diseases”[Title/Abstract]) OR “Chronic Periodontitis”[MeSH Terms]) OR “Chronic Periodontitis”[Title/Abstract]) OR “Alveolar Bone Loss”[MeSH Terms]) OR “Alveolar Bone Loss”[Title/Abstract]) OR Periodontitides[Title/Abstract]) OR Pericementitis[Title/Abstract]) OR Pericementitides[Title/Abstract]) OR “Chronic Periodontitides”[Title/Abstract]) NOT “Chronic Periodontitides”[Title/Abstract]) OR “Periodontitides, Chronic”[Title/Abstract]) OR “Periodontitis, Chronic”[Title/Abstract]) OR “Adult Periodontitis”[Title/Abstract]) OR “Adult Periodontitides”[Title/Abstract]) OR “Periodontitides, Adult”[Title/Abstract]) OR “Periodontitis, Adult”[Title/Abstract]) OR “Disease, Periodontal”[Title/Abstract]) OR “Diseases, Periodontal”[Title/Abstract]) OR “Periodontal Disease”[Title/Abstract]) OR Parodontosis[Title/Abstract]) OR Parodontoses[Title/Abstract]) OR “Pyorrhea Alveolaris”[Title/Abstract]) OR “Periodontitides, Adult”[Title/Abstract]) OR “Periodontal Disease”[Title/Abstract]) OR Parodontosis[Title/Abstract]) OR Parodontoses[Title/Abstract]) OR “Alveolar Bone Losses”[Title/Abstract]) OR “Alveolar Process Atrophy”[Title/Abstract]) OR “Alveolar Process Atrophies”[Title/Abstract]) OR “Alveolar Resorption”[Title/Abstract]) OR “Alveolar Resorptions”[Title/Abstract]) OR “Resorption, Alveolar”[Title/Abstract]) OR “Resorptions, Alveolar”[Title/Abstract]) OR “Bone Loss, Periodontal”[Title/Abstract]) OR “Bone Losses, Periodontal”[Title/Abstract]) OR “Periodontal Bone Losses”[Title/Abstract]) OR “Periodontal Bone Loss”[Title/Abstract]) OR “Periodontal Resorption”[Title/Abstract]) OR “Periodontal Resorptions”[Title/Abstract]) OR “Resorption, Periodontal”[Title/Abstract]) OR “Alveolar Bone Atrophy”[Title/Abstract]) OR “Alveolar Bone Atrophies”[Title/Abstract]) OR “Bone Atrophies, Alveolar”[Title/Abstract]) OR “Bone Atrophy, Alveolar”[Title/Abstract]) OR “Bone Loss, Alveolar”[Title/Abstract]) OR “Bone Loss, Alveolar”[Title/Abstract]) OR “Pyorrhea Alveolaris”[Title/Abstract] |
| SCOPUS | #1 AND #2 |
|  | #1 (TITLE-ABS-KEY(Human*) OR TITLE-ABS-KEY("Modern Man") OR TITLE-ABS-KEY("Man (Taxonomy)") OR TITLE-ABS-KEY("Homo sapiens") AND TITLE-ABS-KEY(Adult*) AND TITLE-ABS-KEY(Cognition*) OR TITLE-ABS-KEY("Cognitive Functions") AND TITLE-ABS-KEY(Memory)) |
|  | #2 ( TITLE-ABS-KEY ( periodontitis ) OR TITLE-ABS-KEY ( "periodontal diseases" ) OR TITLE-ABS-KEY ( "Chronic Periodontitis" ) OR TITLE-ABS-KEY ( "Alveolar Bone Loss*" ) OR TITLE-ABS-KEY ( periodontitides ) OR TITLE-ABS-KEY ( pericementitis ) OR TITLE-ABS-KEY ( pericementitides ) OR TITLE-ABS-KEY ( "Chronic Periodontitides" ) OR TITLE-ABS-KEY ( "Periodontitides, Chronic" ) OR TITLE-ABS-KEY ( "Adult Periodontitis" ) OR TITLE-ABS-KEY ( "Adult Periodontitides" ) OR TITLE-ABS-KEY ( "Periodontal Disease" ) OR TITLE-ABS-KEY ( "Parodontosis" ) OR TITLE-ABS-KEY ( "Parodontoses" ) OR TITLE-ABS-KEY ( "Pyorrhea Alveolaris" ) OR TITLE-ABS-KEY ( "Alveolar Process Atrophy*" ) OR TITLE-ABS-KEY ( "Alveolar Resorption*" ) OR TITLE-ABS-KEY ( "Periodontal Bone Loss*" ) OR TITLE-ABS-KEY ( "Periodontal Resorption*" ) OR TITLE-ABS-KEY ( "Alveolar Bone Atrophy*" ) ) |
|  |  |
| WEB OF SCIENCE | #1 AND #2 |
|  | #1 TS=(Cognition*) OR TS=(Cognitive Function*) OR TS=(Brain) |
|  | #2 TS=(periodontitis) OR TS=(periodontal disease) OR TS=(Periodontitis) OR TS=(Chronic Periodontitis) OR TS=(Alveolar Bone Loss) OR TS=(Adult Periodontitis) OR TS=(Periodontal Bone Loss) OR TS=(Periodontal Resorption*) OR TS=(Alveolar BoneAtrophy*) |
| GOOGLE SCHOLAR | allintitle: Periodontitis and cognitive impairment |
| COCHRANE | #1 Humans and Cognition or "Cognitive Functions" and Memory |
|  | #2 Periodontitis or "periodontal diseases" or "Chronic Periodontitis" or "Alveolar Bone Loss" |
| LILACS | (mh:(Human$)) OR (tw:(“Modern Man”)) OR (tw:(“Man (Taxonomy)”)) OR (tw:(“Homo sapiens”)) OR (tw:(Human$)) AND (mh:(Adult$)) OR (tw:(Adult$)) AND (tw:(Cognition)) OR (tw:(“Cognitive Function$”)) AND (mh:(Memory)) AND (tw:(periodontitis)) OR (tw:(periodontitis)) OR (tw:(“periodontal diseases”)) OR (mh:(“Chronic Periodontitis”)) OR (tw:(“Alveolar BoneLoss”)) |
| OPEN GREY | Periodontitis and cognitive impairment |

**Table Supplementary S3.** Domains and Risk of Bias considered in Risk of Bias evaluation according to Fowkes and Fulton (Fowkes and Fulton, 1991), adapted Almeida et al (Almeida et al., 2017).

| Guideline | Checklist | Description |
| --- | --- | --- |
| Study design apropriate to objectives? | Objective common design | The type of study was marked in the appropriate type of study. If the type of study was appropriate according to the study design was marked as "0" and as "++" if it was not appropriate. |
|  | Prevalence Cross-sectional |  |
|  | Prognosis Cohort |  |
|  | Treatment Controled trial |  |
|  | Cause Cohort, case-control, cross-sectional |  |
| Study sample representative? | Source of sample | The domain was considered (0) in cases of detailed origin, (+) to specified origin of only one group and  (++) in cases of absence of specification of the origin of the groups |
|  | Sampling method | The item was assigned (0) for full description of sampling method, (+) for poor or no description of sample method, with no problem in matching between groups and (++) for poor or no description of sample method, interfering in matching of the groups |
|  | Sample size | A minor problem (+) was considered when the sample was not representative or did not report a sample calculation. To a major problem, (++) was considered when no sample calculation was provided and the number of participants was less than 50 participants, (0) was considered in absence of the above factors. |
|  | Entry criteria/exclusion | A minor (+) problem was attributed when the control group was a case of a history of previous cognitive impairment or diseases that included dementia. In the case of the presence of more than two previously mentioned items was considered a major problem (++). |
|  | Non-respondents | The (0) was attributed when there was no refusal to participation in the study, (+) was assigned when there was refusal, but did not compromise the sample, and (++) when there was refusal and impairment of the sample size. |
| Control group acceptable? | Definition of controls | It was attributed (0) when all characteristics of control group were described, (+) when any information was pendent as the origin of control group, the selection criterions and a different origin between case and control groups and (++) when two or more items described in previously items. |
|  | Source of controls | It was considered (0) when control group was referred, (+) when the origin of groups was different, but with reasons and (++) when the groups present different origins without reasons. |
|  | Matching/randomization | In this item, (0) was assigned to cases of randomized/matched groups, (+) to cases of no description of randomization, but with matching of groups and (++) to no description of randomization or matching. |
|  | Comparable characteristics | It was attributed (0) to matched groups or not matched by the impossibility of being subsequently adjusted and (++) presence of unpaired variables that were not paired or adjusted. |
| Quality of measurements and outcomes? | Validity | It was considered (0) when the evaluation method applied is appropriate; (+) when using a single method, but with appropriate sensitivity with good specificity; (++) when using a single method, without an adequate specificity or good sensitivity. |
|  | Reproducibility | It was considered (0) whether the evaluation methods were well described; (+) when a lack description of any step of the method was presented, for example, the identification of the patients of the groups studied in laboratory samples, evaluations at different times or application of different methods between groups of certain pathology; (++) when two or more of the previous items are present. |
|  | Blindness | The condition of the study participants was considered to be "Blind", in this case being assigned the signal (0), in cases of "not blind" the signal (++) was attributed |
|  | Quality control | It was considered a problem when the evaluators were not calibrated; when ungraduated students carried out the assessment without supervision of a qualified dentist; analysis of periodontitis only radiographic and/or depth of periodontal pockets, to evaluate less than three dental faces or not to mention how many faces were evaluated. When two of these problems were identified, it was considered as a minor problem (+) and major problems (++) if more than two of these characteristics were described. |
| Completeness | Compliance | It was assigned (0) for a sample size that remains the same from the beginning to the end or decreases without compromising the power of the test; (+) for differences in sample size at the end of the study, compromising the power of the test, but with reasons and adjusts; (++) for difference in sample size at the end of the study, compromising the power of the test, without reasons. |
|  | Drop outs | The (0) was scored when there is no loss during the study, (+) when there is withdrawal that involves the inclusion criteria, such as age, sex, (++) when there is withdrawal and it compromises more than one criterion. |
|  | Deaths | This item was scored as Not Applicable (NA), due the type of PECO strategy. |
|  | Missing data | In this item, (0) was assigned to cases of randomized/matched groups, (+) to cases of no description of randomization, but with matching of groups and (++) to no description of randomization or matching. |
| Distorting influences? | Extraneous treatments | In this item, (0) was considered when there were no external influences; (+) when there are external influences, but that does not interfere in the results; (++) when there are external influences and  interferes with the results. |
|  | Contamination | This item was scored as Not Applicable (NA), due the type of PECO strategy. |
|  | Changes over time | In this item, (0) was attributed to data collected in the same time period; (+) to data collected from the control group and the study group at different times that may cause distortions; (++) when the previous item was associated with data from studies already published. |
|  | Confounding factors | A problem was considered in case of the presence of men and women under the age of 45, menopausal, smoker, diabetic and obese women. A "minor" (+) problem was attributed when 1 or 2 of these characteristics were present and a "larger" (++) problem if there were 3 or more. |
|  | Distortion reduced by analysis | It was considered (0) when it cites the adjustments of the covariates that present distortions; (+) when the article report adjustment, but does not say the criteria; (++) when a distortions was identified, without adjustment. |
| Summary questions | **Bias:** | “YES” or "NO" answers were assigned for each question. If the answer is NO at the three questions, the article is considered reliable, with low risk of bias. |
|  | Are the results erroneously biased in certain direction? |  |
|  | **Confounding:** |  |
|  | Are there any serious confusing or other distoring influences? |  |
|  | **Chance:** |  |
|  | Is it likely that the results ocurred by chance? |  |

**References**

Almeida, A., Fagundes, N.C.F., Maia, L.C., and Lima, R.R. (2017). Is There An Association Between Periodontitis And Atherosclerosis In Adults? A Systematic Review. *Curr Vasc Pharmacol*.

Fowkes, F.G., and Fulton, P.M. (1991). Critical appraisal of published research: introductory guidelines. *BMJ : British Medical Journal* 302**,** 1136-1140.

Moher, D., Liberati, A., Tetzlaff, J., and Altman, D.G. (2009). Preferred reporting items for systematic reviews and meta-analyses: the PRISMA statement. *PLoS Med* 6**,** e1000097.
